# Evidence for a role of *Anopheles stephensi* in the spread of drug- and diagnosis-resistant malaria in Africa

**DOI:** 10.1038/s41591-023-02641-9

**Published:** 2023-10-26

**Authors:** Tadele Emiru, Dejene Getachew, Maxwell Murphy, Luigi Sedda, Legesse Alamerie Ejigu, Mikiyas Gebremichael Bulto, Isabel Byrne, Mulugeta Demisse, Melat Abdo, Wakweya Chali, Aaron Elliott, Eric Neubauer Vickers, Andrés Aranda-Díaz, Lina Alemayehu, Sinknesh W. Behaksera, Gutema Jebessa, Hunduma Dinka, Tizita Tsegaye, Hiwot Teka, Sheleme Chibsa, Peter Mumba, Samuel Girma, Jimee Hwang, Melissa Yoshimizu, Alice Sutcliffe, Hiwot Solomon Taffese, Gudissa Aseffa Bayissa, Sarah Zohdy, Jon Eric Tongren, Chris Drakeley, Bryan Greenhouse, Teun Bousema, Fitsum G. Tadesse

**Affiliations:** 1 Armauer Hansen Research Institute, Addis Ababa, Ethiopia; 2 Adama Science and Technology University, Adama, Ethiopia; 3 EPPIcenter program, Division of HIV, ID and Global Medicine, University of California, San Francisco, San Francisco, CA USA; 4 Lancaster Ecology and Epidemiology Group, Lancaster Medical School, Lancaster University, Lancaster, UK; 5 London School of Hygiene and Tropical Medicine, London, UK; 6 Radboudumc, Nijmegen, the Netherlands; 7 U.S. President’s Malaria Initiative, USAID, Addis Ababa, Ethiopia; 8 U.S. President’s Malaria Initiative, Malaria Branch, US Centers for Disease Control and Prevention, Atlanta, GA USA; 9 U.S. President’s Malaria Initiative, USAID, Washington DC, DC USA; 10 U.S. President’s Malaria Initiative, Entomology Branch, US Centers for Disease Control and Prevention, Atlanta, GA USA; 11 Federal Ministry of Health, Addis Ababa, Ethiopia

**Keywords:** Risk factors, Malaria

## Abstract

*Anopheles stephensi*, an Asian malaria vector, continues to expand across Africa. The vector is now firmly established in urban settings in the Horn of Africa. Its presence in areas where malaria resurged suggested a possible role in causing malaria outbreaks. Here, using a prospective case–control design, we investigated the role of *An. stephensi* in transmission following a malaria outbreak in Dire Dawa, Ethiopia in April–July 2022. Screening contacts of patients with malaria and febrile controls revealed spatial clustering of *Plasmodium falciparum* infections around patients with malaria in strong association with the presence of *An. stephensi* in the household vicinity*. Plasmodium* sporozoites were detected in these mosquitoes. This outbreak involved clonal propagation of parasites with molecular signatures of artemisinin and diagnostic resistance. To our knowledge, this study provides the strongest evidence so far for a role of *An. stephensi* in driving an urban malaria outbreak in Africa, highlighting the major public health threat posed by this fast-spreading mosquito.

## Abstract (web summary)

Evidence that a dry-season outbreak of malaria in Dire Dawa, Ethiopia is caused by *Anopheles stephensi* carrying *Plasmodium falciparum* with diagnostic and drug resistance mutations calls for heightened vector surveillance in both urban and rural settings.

## Main

The promising decline in malaria burden has slowed since 2015. This is particularly evident in Africa, the continent that carries the largest malaria prevalence^1^. Malaria control programs in Africa traditionally focus on rural settings, where most infections occur, but malaria is of increasing concern in urban settings^2^. Given the rapid urbanization in Africa^3^, urban malaria transmission can result in a considerable health burden^4^. Urban malaria is classically associated with importation from areas of intense transmission^5^ but can be exacerbated by the adaptation of existing malaria vectors to urban environments^6^ and the emergence of urban malaria vectors such as *Anopheles stephensi*^7^.

**Fig. 1:**
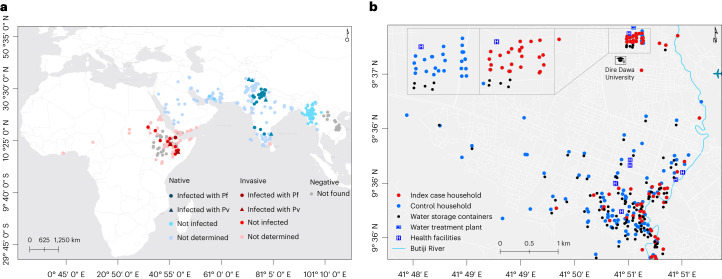
Global distribution of *An. stephensi* and the study location. **a**, The global distribution of *An. stephensi* where it is native (blue) and invasive (red) is shown, together with sporozoite infection detection outcomes where it was found infected and not infected with *P. falciparum* (Pf) and *P. vivax* (Pv). Sites where *An. stephensi* was observed but mosquitoes were not tested for the presence of sporozoites are also shown (not determined). Settings where dedicated entomological surveillance did not detect *An. stephensi* mosquitoes are indicated by gray circles (negative). **b**, The locations of case (red) and control (green) households/dormitories surveyed in this study are shown, together with water storage containers (black), water treatment plant (in the university campus), health facilities (H) and Butiji River in Dire Dawa City. Source: the global map (**a**) was modified on the basis of the malaria threats map^7^ of the WHO. Source data

*An. stephensi* is distinct from other *Anopheles* species that are traditional vectors in (rural) Africa, with its preference for artificial water storage containers that are common in urban settings^8,9^. Native to the Indian subcontinent and the Persian Gulf^10^, *An. stephensi* is now rapidly expanding its geographic range westward (Fig. 1a)^7^. First detected in Africa in Djibouti in 2012 (ref. ^11^), *An. stephensi* has spread across the Horn of Africa; its range now includes Ethiopia (2016)^12^, Sudan (2016)^13^, Somalia (2019)^14^, Eritrea (2022)^15^ and beyond: Yemen (2021)^16^, Kenya (2022)^17^, Ghana (2022)^15^ and Nigeria (2020)^15^. In the Horn of Africa, the vector was found firmly established^18^ and abundantly present in manmade aquatic habitats in the driest months of the year, when endemic vectors such as *An. arabiensis* are largely absent, demonstrating how well adapted the mosquito is to perennial persistence and urban ecology. This poses a risk of year-round malaria transmission. In recognition of the potentially devastating consequences of *An. stephensi* advancing across Africa, the World Health Organization (WHO) urgently requested more data on its distribution and released a strategy to mitigate its spread^19^.

In addition to the invasive *An. stephensi* and widespread high prevalence of insecticide resistance, the Horn of Africa region is disproportionately affected by other emerging biological threats for malaria control, including the emergence of *Plasmodium falciparum* parasites with drug resistance (Uganda^20^, Rwanda^21^ and Eritrea^22^) and histidine-rich protein 2 (*pfhrp2*) and *pfhrp3* gene deletions (Ethiopia^23^, Eritrea^24^ and Djibouti^25^) that could compromise the utility of widely used rapid diagnostic tests (RDTs). Because of its abundant and species-specific expression by *P. falciparum* parasites, histidine-rich protein 2 (HRP2)-based RDTs are commonly used for the diagnosis of *P. falciparum*. Recent reports of expansion of parasites with *pfhrp2*/*pfhrp3* gene deletions and drug resistance, together with a highly efficient invasive mosquito in the region, threaten the major gains documented in recent decades.

In addition to being an efficient vector for both *P. falciparum* and *P. vivax* in its native geographical range^10^, *An. stephensi* was recently confirmed as being susceptible to local parasites in Ethiopia (Fig. 1a)^9,18^ and a resurgence of malaria was reported in Djibouti following its detection in 2012 (ref. ^26^), although direct evidence for a role of *An. stephensi* in this resurgence was unavailable. In this Article, following a report of a dry-season upsurge in malaria cases in Dire Dawa City, Ethiopia, where *An. stephensi* was recently documented^8^, we prospectively investigated its role in malaria transmission through responsive epidemiological and entomological surveillance (Fig. 1b).

## Results

### Malaria outbreaks in Dire Dawa City and its university

**Fig. 2:**
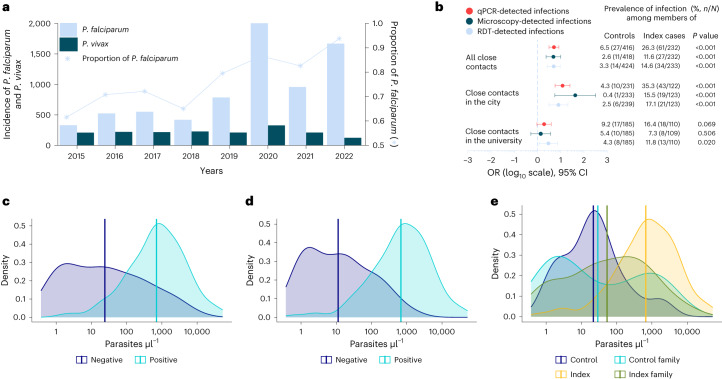
Temporal trends in malaria burden and parasite density distributions in Dire Dawa. **a**,**b**, Malaria trends using DHIS2 data (**a**) are shown, with the prevalence and odds of detecting additional infections in close contacts of cases compared with controls in Dire Dawa, separately for all close contacts, contacts in the city and the university (**b**). ORs were obtained from univariate logistic regression, with diagnostic test results as outcome and site as predictor. Univariate logistic models were fitted for each diagnostic test. ORs are shown on a log_10_ scale (*x* axis), together with their 95% CI bars and respective *P* values (estimated from Wald test). Numbers to the right of the forest plot indicate the proportion of positive cases by respective diagnostic test (color coded and embedded in the figure) among control and index household/dormitory members. **c**–**e**, Parasite density per microliter distributions and their respective averages determined by 18S-based qPCR among HRP2-based RDT-positive (*n* = 113) and RDT-negative (*n* = 88) infections (**c**) and microscopy-positive (*n* = 129) and microscopy-negative (*n* = 71) infections (**d**) are shown, together with distribution among index cases (*n* = 99), contacts of index cases (*n* = 61), controls (*n* = 14) and contacts of controls (*n* = 27) (**e**). Source data

Clinical malaria incidence data (diagnosed by microscopy) collected from public and private health facilities (*n* = 34) showed an overall statistically significant trend of increasing number of malaria-positive cases between 2019 and 2022 (Mann–Kendall statistical test *τ* = 0.42, *P* < 0.001). A 12-fold increase was observed (Extended Data Table 1 and Supplementary Fig. 1) for malaria incidence in Dire Dawa during the dry months (January–May) of 2022 (2,425 cases) compared with 2019 (205 cases). A similar increasing trend was observed using District Health Information System 2 (DHIS2) data (Fig. 2a and Supplementary Fig. 1). Patients reported at both public and private health facilities, with the latter contributing to 15.8% of patients diagnosed for malaria in the past 4 years with an increasing trend from 17.7% in 2019 to 25.9% in 2021, which later declined to 5.7% during the outbreak (2022). In 2022, 76% of all reported malaria cases originated from only three public health facilities: Dire Dawa University (DDU) students’ clinic (42%), Sabiyan Hospital (19%) and Goro Health Center (15%). At DDU campus, 94% (1,075 out of 1,141) of clinical malaria episodes occurred in the male student population living in the university’s single-sex dormitories.

We conducted a prospective case–control study to identify risk factors associated with this sudden rise in malaria in the city (Goro Health Center) and DDU (Fig. 1b). In the city we recruited 48 microscopy-confirmed febrile malaria cases plus 125 case household members and 109 febrile controls without microscopy-confirmed malaria who had attended the same clinic within 72 h, plus 241 control household members. At DDU we recruited 53 students with clinical malaria and 110 dorm-mates and 80 uninfected febrile students with 186 dorm-mates. Details of individual and household characteristics are presented in Table 1. Both index cases and controls were febrile either at the time of recruitment or within 48 h (self-reported) before attending the clinics. Family members/dorm-mates were recruited irrespective of symptoms. Fever was detected in a minority of recruited family/dormitory members of the controls (1.4%, 6 out of 424) and index cases (6.0%, 14 out of 233; Extended Data Table 2). The responsive case–control study unit was household/dormitory; no plausible risk factors were defined a priori, and neither a sex/gender nor *Plasmodium* species stratification was considered in the study design. The outbreak at the university campus occurred at a fine spatial scale (20 dormitory buildings in a 45,450-m^2^ area); the dormitories affected by malaria were occupied by male students only (Extended Data Table 1). Despite Dire Dawa being historically coendemic for *P. falciparum* and *P. vivax*, the proportion of malaria cases due to *P. falciparum* increased from 61% in 2015 to 93% in 2022 (Fig. 2a). All index cases that we recruited (*n* = 101) and the additional infections detected (*n* = 102) in this study were found to be *P. falciparum*, with the exception of two *P. vivax* infections detected by 18S-based quantitative polymerase chain reaction (qPCR). *Plasmodium* infection was detected in 14 controls by 18S-based qPCR. The parasite density in these infections—which were all *P. falciparum*—was very low (median parasitemia was 21 parasites µl^–1^) and thus lies below the detection limits of conventional diagnostics. Only two of these infections had parasitemia >100 parasites µl^–1^ (278 and 1,822 parasites µl^–1^).

**Table 1:** Summary statistics of individual- and household-level characteristics for members of the cases and controls in the two settings in Dire Dawa

Characteristics	City		University	
	Cases	Controls	Cases	Controls
Individual characteristics, % (*n*/*N*)				
Number of participants (*n*)	173	350	163	266
Malaria incidence	42.1 (72/173)	2.3 (8/350)	42.9 (70/163)	5.3 (14/266)
Fever (axillary temperature ≥37.5 °C)	0.9 (1/110)	1.(2/186)	10.6 (13/123)	1.7 (94/238)
Male sex	47.9 (83/173)	45 (157/349)	100 (163/163)	100 (266/266)
Age (years), median (IQR)	23 (14,35)	22 (11,35)	22 (21,23)	21 (20,22)
Travel history past month	9.3 (16/173)	9.5 (33/349)	9.8 (16/163)	6.4 (17/266)
Long-lasting insecticide-treated nets use	41.9 (67/160)	50.9 (169/332)	41.4 (67/162)	41.7 (105/252)
Use of aerosol insecticide sprays	12.3 (19/155)	23.7 (75/316)	0.0 (0/160)	0.4 (1/259)
Wood smoke in the house the previous night	25.3 (39/152)	21.3 (68/320)	0.0 (0/163)	0.0 (0/266)
Household characteristics, % (*n*/*N*)				
Number of households (*n*)	48	109	53	80
Larval positivity within 100-m radius around household	14.6 (7/48)	4.6 (5/109)	17.0 (9/53)	5.0 (4/80)
Adult *An*. *stephensi* presence (indoor/outdoor)	2.1 (1/48)	0.0 (0/109)	13.2 (7/53)	10.0 (8/80)
*An. stephensi* positivity (larvae and/or adults)	16.7 (8/48)	4.6 (5/109)	30.0 (16/53)	15.0 (12/80)
Livestock presence	31.9 (15/47)	38.3 (36/94)	0.0 (0/53)	0.0 (0/80)
Average distance to river (m)	666.9	488.9	385.3	394.8
Average distance to artificial containers (m)	688.7	661.5	68.5	65.2
Eave opened	4.7 (2/43)	6.2 (6/97)	54.9 (28/51)	52.1 (37/71)
Modal water body type	Stream	Stream	Pond	Pond
Water body presence within 100-m radius around household	47.9 (23/48)	44.0 (48/109)	96.2 (51/53)	98.8 (79/80)
Insecticidal residual spray in the past 12 months	2.3 (1/44)	0.0 (0/104)	26.9 (14/52)	13.2 (10/76)

*n*/*N*, counts over totals for each characteristic are shown in brackets.

### Mosquito exposure and infection prevalence in malaria contacts

The results obtained from case–control analysis showed that members of index cases and controls had different levels of mosquito exposure (Extended Data Table 3). In entomological surveillance, all households and dormitories were surveyed for adult mosquitoes (indoors, outdoors and in animal shelters if present) and immature stages of *Anopheles* in waterbodies present within a 100-m radius. Members of a case household/dormitory were more likely to be living close to *An. stephensi-*positive sites, defined as the presence of larvae within a 100-m radius from the household/dormitory (odds ratio (OR) 5.0, 95% confidence interval (CI) 2.8–9.4, *P* < 0.001), to adult mosquito resting sites (OR 1.9, 95% CI 0.9–4.0, *P* = 0.068) or to natural/manmade waterbodies in general (OR 1.6, 95% CI 1.2–2.2, *P* = 0.002). The odds of using an aerosol insecticide spray were 58% lower among members of index cases compared with controls (OR 0.42, 95% CI 0.23–0.72, *P* < 0.001).

In the city, *P. falciparum* qPCR-detected infections were significantly more common (OR 12.0, 95% CI 5.8–25.1, *P* < 0.001; Fig. 2b) among case household members (35.3%, 43 out of 122) than control household members (4.3%, 10 out of 233), with a similar trend for microscopy- (OR 42.4, 95% CI 5.6–320.8, *P* < 0.001) and RDT-detected infections (OR 8.0, 95% CI 3.1–20.4, *P* < 0.001). At DDU, despite all students living in close proximity (20 buildings in a 45,450-m^2^ area), dorm-mates of malaria cases were three times more likely (OR 3.0, 95% CI 1.2–7.4, *P* = 0.020; Fig. 2b) to be *P*. *falciparum* positive by RDT (11.8%, 13 out of 110) compared with dorm-mates of controls (4.3%, 8 out of 185). One-quarter of microscopy-positive infections (34 out of 136) were negative by HRP2-based RDT (sensitivity 75.0, 95% CI 72.2–77.8, specificity 97.0, 95% CI 95.9–98.1; Extended Data Table 4), with different proportions of HRP2-based RDT-negative infections in the city (10.3%, 7 out of 68) and the university (39.7%, 27 out of 68). HRP2-based RDTs are those most commonly used in the diagnosis of *P. falciparum* in the area. Recent reports of expansion of parasites with *pfhrp2*/*pfhrp3* gene deletion threaten the important role of these RDTs in the diagnosis of malaria. qPCR detected considerably more infections, with the likelihood of infections being missed by RDT (Fig. 2c) or microscopy (Fig. 2d) being dependent on parasite density and, for RDT, *pfhrp2* gene deletion status (Extended Data Table 5). Parasite densities were higher in RDT-positive infections (geometric mean 702 parasites µl^–1^, 95% CI 495–997) than RDT-negative infections (geometric mean 24, 95% CI 14–42, *P* < 0.001). Similarly, parasite densities were higher in microscopy-positive infections (683 parasites µl^–1^, 95% CI 488–956) than in microscopy-negative infections (11 parasites µl^–1^, 95% CI 7–19, *P* < 0.001; Extended Data Table 5). Median parasite density (per microliter) as determined by qPCR for infections that were RDT negative but microscopy positive was 357,236 (interquartile range (IQR) 51,440–1,790,966, *n* = 31), strongly suggestive of *pfhrp2*/*pfhrp3* gene deletion in these infections. Parasite density distributions were not different between university students (geometric mean 158 parasites µl^–1^, 95% CI 94–265) and city residents (163 parasites µl^–1^, 95% CI 91–291, *P* = 0.132; Supplementary Fig. 2). As expected, parasitemia was higher in index cases (geometric mean 669 *P. falciparum* parasites µl^–1^, 95% CI 442–1012; Fig. 2e) compared with malaria-infected controls (21 parasites µl^–1^, 95% CI 7–67, *P* < 0.001), malaria-infected control family members (29 parasites µl^–1^, 95% CI 9–97, *P* = 0.005) and malaria-infected index family members (53 parasites µl^–1^, 95% CI 27–107, *P* < 0.001).

### *An. stephensi* dominates and carries *P. falciparum* sporozoites

*Anopheles* larvae were detected in 3% (26 out of 886) of aquatic habitats, which were either artificial (*n* = 17) or natural (*n* = 9). *An. stephensi* was the only species detected in artificial containers (*n* = 414 larvae), of which the majority were metal and plastic barrels and jerrycans, and was the predominant species detected at stream edges (57% larvae, 160 out of 280; Extended Data Table 6). Adult *Anopheles* spp. mosquitoes were detected in the majority of examined animal shelters (18 out of 24), water storage tankers (4 out of 4), manholes (7 out of 7), inside (22 out of 508) and outside (7 out of 305) the index and control households/dormitories using Prokopack aspirators, with nearly all identified as *An. stephensi* (97%, 599 out of 618; Extended Data Table 7). All mosquitoes morphologically identified as *An. stephensi* and tested molecularly (*n* = 90) were confirmed to be this species, except four for which the *ITS2*-based PCR experiment failed (Supplementary Fig. 3)—which may have been the result of loss of genetic material during extraction. Fully engorged adult-caught *An. stephensi* (195 out of 599) and *An. gambiae* (5 out of 16) mosquitoes (Extended Data Table 8) were tested for bloodmeal sources in a multiplex PCR assay that amplifies the cytochrome-b gene: for cow, dog, goat and human. Goats or cows were the main recent bloodmeal sources of *An. stephensi* (98%, 96 out of 98) and *An. gambiae* s.l. (80%, 4 out of 5), but only *An. stephensi* (2 out of 98) had recently fed on humans. Bloodmeal source was undetermined for one-half (*n* = 96) of fully engorged (*n* = 199) *An. stephensi* mosquitoes tested in this study. *P. falciparum* sporozoites, indicative of transmission following natural blood feeding, based on sporozoite and PCR-based detection, were confirmed only in *An. stephensi* (0.5%, 3 out of 599).

### Overlapping clusters of *P. falciparum* and *An. stephensi* abundance

**Fig. 3:**
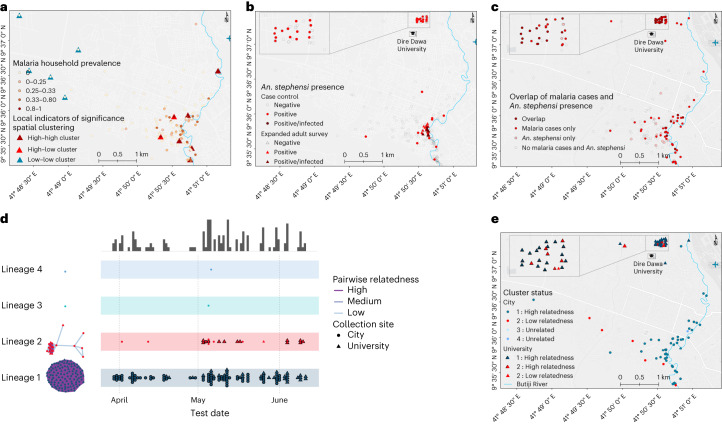
Spatial distribution and clustering of *P. falciparum* parasites and *An. stephensi*. **a**–**c**, Statistically significant evidence for global spatial clustering of household *P. falciparum* infections prevalence (**a**) and *An. stephensi* mosquitoes (**b**) and an overlap between the two (**c**) were observed. Eleven clusters of households were found (A) in the city (*P* < 0.05) by one-sided local Anselin Moran’s *I* test (pseudo *P* values calculated from 9,999 random permutations): high–high (*n* = 6) whereby households had high *P. falciparum* prevalence, low–low clusters (*n* = 5) whereby households had low *P. falciparum* prevalence, and high–low outlier clusters (*n* = 2) whereby high *P. falciparum* prevalence households were surrounded by low *P. falciparum* prevalence households, or vice versa. Locations of *An. stephensi* mosquitoes found infected (*n* = 3) are shown in dark-red circles and triangles (**b**). **d**,**e**, A map displaying case incidence colored by genetic cluster (lineage 1 in blue and lineage 2 in red) are shown along with timelines that cases were identified (**d**) and their spatial distribution (**e**). Source data

Spatial analysis of *P. falciparum* infection localities within the city demonstrated significant evidence for clustering (global Moran’s *I* test 0.020; *P* < 0.001; Fig. 3a) in the study area, and 11 significant clusters of *P. falciparum* infection (detectable by microscopy and/or RDT) were detected. *An. stephensi* larvae and/or adult mosquitoes were more often detected near index cases (14.9%) than controls (4.3%, *P* = 0.020; Fig. 3b), and this overlapped with clusters of *P. falciparum* infections (Fig. 3c). Sporozoite-infected mosquitoes were also found in close proximity (Fig. 3b). In the city, clusters of households with higher infection prevalence were all situated within 200 m of Butiji River.

### *An. stephensi* presence is strongly linked with *P. falciparum* positivity

We next evaluated risk factors for being infected with *P. falciparum* (Table 2). Male sex (OR 3.0, 95% CI 1.7–5.4, *P* = 0.001) and being above 15 years of age (OR 4.3, 95% CI 1.2–15.7, *P* = 0.029) were risk factors associated with *P. falciparum* infection positivity, while using aerosol insecticide sprays was found protective from malaria (OR 0.3, 95% CI 0.1–0.8, *P* = 0.016). The results further show that those individuals residing in households/dormitories with *An. stephensi* positivity (larvae/adult/indoor/outdoor) had a higher risk of malaria infection (OR 3.7, 95% CI 1.7–6.5, *P* < 0.001) compared with individuals in households/dormitories where *An. stephensi* was not detected.

**Table 2:** Results from a multi-level logistic regression model with nested random effects for being infected with *P. falciparum* in Dire Dawa City

Factors	Category	Proportion parasite positive, % (*n*/*N*)	Unadjusted		Adjusted	
			OR (95% CI)	*P* value	OR (95%CI)	*P* value
Sex	Female (Ref.)	10.3 (29/281)				
	Male	20.2 (134/665)	2.3 (1.4–3.9)	0.001	3.0 (1.7–5.4)	<0.001
Age in years	<5 years (Ref.)	5.3 (3/57)				
	5–15 years	16.4 (18/110)	4.1 (1.1–15.3)	0.036	3.7 (0.9–14.9)	0.071
	Above 15 years	15.2 (142/779)	3.8 (1.1–13.0)	0.035	4.3 (1.2–15.7)	0.029
*An. stephensi* larvae and/or adult presence	Absent (Ref.)	15.3 (132/269)				
	Present	36.5 (31/85)	3.2 (1.8–5.8)	<0.001	3.3 (1.7–6.5)	<0.001
Natural waterbody presence	Absent (Ref.)	11.2 (32/269)				
	Present	19.7 (133/677)	2.0 (1.2–3.3)	0.007	1.8 (0.9–3.4)	0.089
Usage of aerosol insecticide spray	Not using (Ref.)	18.6 (147/790)				
	Using	7.4 (7/95)	0.3 (0.1, 0.8)	0.013	0.3 (0.1–0. 8)	0.016

Results from univariate and multivariate generalized linear mixed model. Study site, household and case/control were included as nested random effects after adjusting sex and age for study sites. Only those risk factors with *P* values lower than 0.1 in univariate analyses were considered for multivariate analysis. The estimated variance between nested household and case/control for the final model was 1.06, which corresponds to intra-cluster correlation of 0.24. Ref., reference category.

### Parasites with signatures of artemisinin and diagnostic resistance

We attempted to sequence 18S qPCR-positive samples and of these the sequencing was successful for 71% (*n* = 131) of the samples. All blood samples were collected from patients before treatment was provided, and thus represent the composition of parasites in the blood. Genotyping of 131 infections at 162 microhaplotype loci by amplicon sequencing uncovered that 90% of infections were monoclonal and nearly all were closely related to other detected infections, with 98% falling into one of two distinct, nearly clonal lineages. Lineage 1 was the most common, almost completely homogeneous, observed throughout the study period, and distributed widely throughout both study sites (Fig. 3d,e and Table 3). Lineage 2 accounted for 15% of infections and contained some genetic diversity, with only 13 of 20 infections highly related to each other. Highly related infections within lineage 2 were not detected until May 2022, with most (11/13) detected at DDU (Fig. 3c). Infections within dormitories were not restricted to a single lineage; half (7/14) of all dormitories with more than one infection had infections from both lineages detected. Of concern was that 14 out of 20 lineage 2 infections carried the R622I mutation in the *kelch13* gene—which has been associated with reduced susceptibility to artemisinins in Eritrea^22^—along with evidence of *P. falciparum pfhrp2* and *pfhrp3* gene deletions—which are associated with false negativity of HRP2-based RDTs. Consistent with evidence of deletions of these genes, the majority of lineage 2 parasites (70.0%, 14/20) tested negative on HRP2-based RDT but were positive by microscopy. Lineage 1 infections did not contain *pfhrp2* deletions, most were detectable by RDT (71.6%, 78/109), and only 2.8% (*n* = 3) contained the *kelch 13* R622I mutation, but all had evidence of *pfhrp3* deletions and the quintuple mutation in *pfdhfr* and *pfdhps* associated with antifolate resistance. Of the successfully sequenced microscopically detectable but RDT-negative infections (*n* = 24), some were found to be *pfhrp2* and *pfhrp3* double gene deleted (37.5%, 9/24) while the rest were only *pfhrp3* gene deleted (62.5%, 15/24). Interestingly, most infections from lineage 2 containing the R622I mutation (11/14) exhibited incomplete antifolate resistance, lacking the *pfdhfr* 59 mutation. A single monoclonal infection with low relatedness within lineage 2 showed unique features: elevated *pfmdr* copy number, heterozygous for the *pfmdr1* 184 mutation, while being the only infection with a wild-type *pfcrt* genotype. There was no significant association between lineage 1 and lineage 2 with self-reported uptake of vector control measures (bed net utilization, insecticide residual spray and repellent use), travel history, age, sex, educational level, occupation or infection detection by microscopy (Extended Data Table 9). In contrast, a larger proportion of lineage 2 infections were undetected by RDT, as described above. These data, showing primarily clonal transmission of two distinct parasite lineages that did not intermix, are consistent with increased transmission occurring on the background of an exceedingly small parasite population, with more recent spread of a parasite lineage containing mutations that are concerning for drug and diagnostic resistance.

**Table 3:** Summary of diagnostic results and drug resistance genotype prevalence stratified by lineage, clonality and within-lineage relatedness

Lineage	Overall	1			2				
Subset		All	Monoclonal	Polyclonal	All	Monoclonal	Polyclonal	High relatedness	Low relatedness
*N*	131	109	105	4	20	13	7	13	7
RDT^+^ (%)	84 (64.1)	78 (71.6)	78 (74.3)	0 (0)	6 (30)	3 (23.1)	3 (42.9)	2 (15.4)	4 (57.1)
Microscopy^+^ (%)	97 (74)	82 (75.2)	82 (78.1)	0 (0)	15 (75)	11 (84.6)	4 (57.1)	11 (84.6)	4 (57.1)
*pfhrp2* deleted (%)	12 (9.2)	0 (0)	0 (0)	0 (0)	12 (60)	11 (84.6)	1 (14.3)	11 (84.6)	1 (14.3)
*pfhrp3* deleted (%)	127 (96.9)	109 (100)	105 (100)	4 (100)	16 (80)	12 (92.3)	4 (57.1)	13 (100)	3 (42.9)
qPCR geometric mean, parasite µl^−1^ (IQR)	220 (48–1,800)	210 (51–1,700)	240 (76–1,700)	6.1 (3.4–17)	460 (87–3,400)	950 (280–2,900)	120 (2.1–6,300)	470 (280–2,200)	440 (19–20,000)
*pfk13* 622I (%)	17 (13.4)	3 (2.8)	3 (2.9)	0 (0)	14 (73.7)	9 (75)	5 (71.4)	12 (100)	2 (28.6)
*pfdhps* 437/540 (%)	128 (99.2)	107 (100)	103 (100)	4 (100)	19 (95)	12 (92.3)	7 (100)	13 (100)	6 (85.7)
*pfdhfr* 51/108 (%)	128 (99.2)	107 (100)	103 (100)	4 (100)	19 (95)	12 (92.3)	7 (100)	13 (100)	6 (85.7)
*pfdhfr* 59/108 (%)	116 (89.9)	107 (100)	103 (100)	4 (100)	7 (35)	2 (15.4)	5 (71.4)	1 (7.7)	6 (85.7)
*pfdhfr* 51/59/108 (%)	116 (89.9)	107 (100)	103 (100)	4 (100)	7 (35)	2 (15.4)	5 (71.4)	1 (7.7)	6 (85.7)
*pfdhps* 437/540 + *pfdhfr* 51/59/108 (%)	115 (89.1)	107 (100)	103 (100)	4 (100)	6 (30)	1 (7.7)	5 (71.4)	1 (7.7)	5 (71.4)
*pfcrt* CVIET^a^ (%)	130 (99.2)	109 (100)	105 (100)	4 (100)	19 (95)	12 (92.3)	7 (100)	13 (100)	6 (85.7)
*pfmdr1* 184Y (%)	1 (0.8)	0 (0)	0 (0)	0 (0)	1 (5)	1 (7.7)	0 (0)	0 (0)	1 (14.3)

Lineage 3 (monoclonal) and lineage 4 (polyclonal) infections were *pfhrp3* deleted, negative both by microscopy and RDT, and mutated for all drug resistance variants (except *pfk13* 622I and *pfmdr* 184Y). ^a^*pfcrt* CVIET, *pfcrt* 72Cys–73Val–74Ile–75Glu–76Thr. Proportions are shown within brackets.

## Discussion

Our findings raise concern about urban malaria associated with the presence of *An. stephensi*. First detected in 2018 in Dire Dawa^8^, *An. stephensi* is now perennially present in the city and was found infected with *P. falciparum*^18^. In 2014, no *Anopheles* developmental stages were detected in containers in Dire Dawa^27^, supporting the notion of its recent introduction in the area. In the years following its first detection (between 2019 and 2022), a 12-fold increase in malaria incidence that was predominantly *P. falciparum* was observed in the city. The spatial overlap and association between malaria infection and the presence of *An. stephensi*, the detection of sporozoites in adult mosquitoes and the clonal propagation of parasites that we report here provide the strongest evidence so far for a role of *An. stephensi* in driving an urban malaria outbreak in Africa. This, to our knowledge, is the first direct evidence of the role of *An. stephensi* in transmitting malaria in Africa and corroborates recent reports from Djibouti of exponential increases in malaria cases in the years following detection of the species^26^.

The outbreak in the university campus was localized and the dormitories affected by malaria were occupied by male students only. However, in the population of Dire Dawa City, male sex and older age were predictors of malaria positivity. Higher parasite prevalence in males compared with females has been reported in Ethiopia^28^, other African countries^29^ and Brazil^30^, and is commonly described in South East Asia^31^. Common explanations are increased risk due to employment and sociobehavioral factors (for example, use of preventive measures, sleeping times and forest work). There may be other behavioral differences between males and females involving crepuscular activities consistent with biting times for *An. stephensi*, which is exophilic and exophagic^32^. In our setting, chewing khat outdoors is done predominately by men^33^ again increasing exposure to vectors. There is limited evidence for sex-associated biological differences in infection acquisition or infection consequences, with the exception of the well-established role of pregnancy in malaria risk^34^. The recently described longer infection duration in males compared with females^35^ suggests that there may be differences in infection kinetics/responses to infections between sexes that may in turn impact the epidemiology of malaria infection.

Interestingly, this outbreak only involved *P. falciparum* infections despite the co-occurrence of *P. vivax* in the region. We previously demonstrated that *An. stephensi* is highly susceptible to Ethiopian *P. vivax* isolates^9^ and an increase in *P. vivax* cases coincided with a rise in *An. stephensi* mosquitoes in Djibouti^26^. Epidemiological circumstances at the start of the outbreak, notably the extent of the human infectious reservoir for *Plasmodium* infections, may have been more favorable for *P. falciparum* in our setting. In sympatric settings, it is well known that *P. falciparum* is more prone to epidemic expansion than *P. vivax*^36,37^. There is a large and increasing body of evidence (including our own work from Ethiopia)^38,39^ showing that asymptomatic *P. falciparum* infections can be highly infectious to mosquitoes and that the level of infectivity depends on the circulating parasite biomass (that is, parasite density in asymptomatic carriers). Related studies on the human infectious reservoir for *P. falciparum* have also demonstrated that a limited number of individuals, sometimes with asymptomatic infections, may be highly infectious to mosquitoes^39^. This hypothesis is supported by the limited genetic diversity of parasites detected in this study. We speculate that, at the start of the outbreak, the asymptomatic infectious reservoir for *P. falciparum* was larger than for *P. vivax* and that a small number of infected individuals may have been responsible for initiating the current outbreak. The continued increase in the proportion of *P. falciparum* infections between 2015 and 2022 in Dire Dawa and the timing of the outbreak supports this notion. Although sporozoite rates are difficult to compare between sites, times and species, since they depend on many factors including mosquito age and survival, the 0.5% *P. falciparum* sporozoite positivity that we observed is similar to that observed previously in *An. stephensi* in Dire Dawa and other areas in Ethiopia^18^ as well as sporozoite rates in *An. arabiensis*, a native malaria vector in Ethiopia^40^. We consider a comparison with other areas with markedly different parasite populations and transmission intensity less relevant although sporozoite rates of *An. stephensi* in Afghanistan (0.8%) and India (0.6%) are in the same range as we observed^41^. Higher sporozoite rates are more likely to be associated with sustained endemicity (with entomological inoculation rate >1) and are typically associated with microscopy parasite prevalence between 10% and 40% (ref. ^42^). Continuous entomological and clinical surveillance would provide further evidence if this was the case in Dire Dawa. In contrast, asymptomatic *P. vivax* infections have typically too low parasite densities to infect mosquitoes^38,43^. Since *P. vivax* sporozoites have been detected in *An. stephensi* mosquitoes previously from the same setting^18^, it is possible that future malaria outbreaks caused by *An. stephensi* would also involve *P. vivax*.

The trends in increased parasite carriage among individuals living in proximity of malaria cases were most apparent for conventional diagnostics (RDT and microscopy) but not for qPCR. This is probably to reflect the age of infections with recent infections (that is, acquired during the outbreak under examination) being more likely to be of higher parasite density while low-density infections that are detectable by qPCR to mainly reflect old infections that may have been acquired many months before the study^44^. Historical transmission levels influence the size of the submicroscopic reservoir through acquired immunity^45^. As Dire Dawa was previously endemic^46^, some low-density infections may persist and affect the interpretation of the extent of the outbreak. The relatively high-density (microscopy-detected) asymptomatic infections provided a better description of the current outbreak^38^.

In addition to the role for the invasive *An. stephensi*, two other biological threats for the control of *P. falciparum* were identified in our study: drug resistance and diagnostic resistance. The high prevalence of parasites with the R622I mutation in the *kelch13* gene is of particular concern. Although it should be noted that parasite strains were not directly tested for resistance ex vivo in the current study, a recent study identified this as a variant linked with partial drug resistance in Eritrea^22^. Following the first report in 2016 from northwest Ethiopia^47^, parasites carrying the R622I variant are reported to be expanding in the same setting^48^, more widely in the country^49^ and elsewhere in the Horn of Africa^50^. In addition to evidence for artemisinin-resistant parasites, mutations conferring chloroquine and antifolate resistance were common in the parasite population responsible for this outbreak. Similarly, *pfhrp2* and *pfhrp3* gene deletions with phenotypic evidence of RDT negativity were detected in our study. Despite its first report from Peru^51^, the Horn of Africa (Ethiopia^23^, Eritrea^24^, Sudan^52^, South Sudan^53^ and Djibouti^25^) is disproportionately affected by diagnostic challenges of infections with *pfhrp2*/*pfhrp3* deletions. Co-occurrence of parasites with *pfhrp2*/*pfhrp3* gene deletions and the R622I mutation was recently reported from other sites in Ethiopia^49^. So far, no evidence exists if the drug resistance conferring *kelch13* mutation (R622I) and *pfhrp2*/*pfhrp3* gene deletions have co-evolved in the region or if this is a matter of coincidence. Even without the evidence of co-evolution, the convergence of the three biological threats (*kelch13* mutation, *pfhrp2*/*pfhrp3* gene deletion, and *An. stephensi* playing a role in sustaining transmission of these parasites) is concerning for the region and the entire continent at large.

In this study we concurrently examined parasite carriage and spatial clustering in humans and mosquitoes as well as genetic linkage analysis to demonstrate a highly plausible role for *An. stephensi* in an outbreak of *P. falciparum* infections that carry diagnostic and drug resistance markers in Ethiopia. Our data, demonstrating *An. stephensi* being abundant both in artificial and natural aquatic habitats in the driest months of the year, highlight how well-adapted the mosquito is to perennial persistence and urban ecology. While our outbreak investigation was performed shortly after the mosquito species was first detected in the area^8^, routine vector surveillance was sparse and we cannot draw firm conclusions on the timing of *An. stephensi* introduction in the area. Additionally, limited sensitivity of methodologies for sampling exophagic adult mosquitoes may have resulted in an underestimation of mosquito exposure and reduced precision of sporozoite prevalence estimates. Common adult mosquito collection methods have limited sensitivity for this invasive exophilic/exophagic species. Enhanced surveillance in this study revealed outdoor resting sites (manholes, water storage tankers and animal shelters) that offer opportunities for targeted vector control and highlight the behavioral plasticity of this invasive mosquito which makes it less amenable to conventional control approaches. Our data on the use of protective measures (for example, repellents) were insufficiently detailed to explore how effective these measures are against *An. stephensi*. Future studies should address this. Considering the very high level of resistance of *An. stephensi* to the major insecticides in Ethiopia^18,54^, the repellent effect of the aerosol sprays is one explanation for the protective association observed in this study^55^. Most sprays contain repellents such as *N*,*N*-diethyl-meta-toluamide (DEET) or permethrin. Permethrin and DEET have strong repellent effects on both *Plasmodium*-infected and uninfected *An. stephensi* mosquitoes^55^.

In terms of public health consequences, the spread of *An. stephensi* in rapidly expanding urban settings could pose a challenge to malaria control programs in Africa for four main reasons: (1) its year round persistence due to its ability to exploit manmade containers that are abundantly present in rapidly expanding urban settings; (2) its ability to evade standard vector control tools given its unique ecology and resistance to many of the currently available insecticides; (3) its ability to efficiently transmit both *P. falciparum* and *P. vivax* in the region; and (4) its confirmed role in sustaining the transmission of drug and diagnostic resistant parasites demonstrated in this study that highlights a concerning convergence of biological threats for malaria control in the Horn of Africa and beyond. There is an urgent need for intensified surveillance to identify the extent of the distribution of this vector and to develop and implement tailored control measures. While there is an increasing body of high-quality evidence of the spread of *An. stephensi* across the African continent, pragmatic studies on how to address this novel malaria threat are largely absent. Given increasing reports of *An. stephensi* in West and East Africa, the time window during which elimination of this mosquito from (parts of) Africa is possible is rapidly closing.

## Methods

### Description of the study area

Dire Dawa, located 515 km southeast of Addis Ababa (capital of Ethiopia) and 311 km west of Djibouti, is a logistics hub for transportation of goods and cargo (Fig. 1b). Of its total population (445,050), 74% live in an urban area which is only 2.3% of the 1,288 km^2^ Dire Dawa City administrative land (UN-HABITAT, 2008). The area has a warm and dry climate with low level of precipitation (annual average rainfall of 624 mm), and an annual temperature ranging from 19 °C to 32 °C. Malaria incidence has historically been low (an annual parasite clinical incidence of <5 per 1,000 people between 2014 and 2019), with strong seasonality (August to November being the peak season), and sympatric *P. falciparum* and *P. vivax* infections.

We obtained public health data, collected through the District Health Information System 2 (DHIS2), to analyze the trend in malaria cases between 2015 and 2022. In the Ethiopian malaria case management guideline, microscopy is recommended for diagnosis at the health center level and above. RDTs are recommended to be used only at the health post level by community health extension workers, in rural settings. In all of the facilities located in Dire Dawa, microscopy was used for diagnosis. The DHIS2 data do not capture cases detected at private health facilities. The recent ‘Global framework for the response to malaria in urban areas’ by the WHO^4^ states that “In some urban settings, the private sector is a major source of malaria diagnosis and treatment. However, it is poorly integrated into the surveillance system”. To give context on how much is being managed by the private sector in Dire Dawa, we have collected 4 years of data (January 2019 to May 2022) from 34 out 39 health facilities (both private and public) that are located within the city administration. This included 2 public and 5 private hospitals, 15 health centers (funded publicly) and 17 clinics (private). Some private clinics (*n* = 5) refused to provide data or provided incomplete data. Goro Health Center and DDU students’ clinic were selected for the current study based on the highest number of cases they reported before the start of the study (January–February 2022). In fact, together, the two health facilities reported 56% of the total cases in the city in 2022 (January–May). As in all public universities in Ethiopia, students live within campus with full and shared accommodation provided by the government. At DDU, an average of six students of the same sex and year of study share a dormitory on a three-story building that has an average of 67 dormitories. Routine healthcare service is provided in a university dedicated students’ clinic.

### Study design and procedure

To ascertain the effect of exposure to *An. stephensi* on malaria, we employed a case–control study where identification of patients was done prospectively to capture co-occurrent characteristics in terms of exposure and risk factors. We recruited consecutive patients with criteria described below in a 1:2 ratio (one case:two controls) unmatched study design. Study protocol was approved by the Institutional Ethical Review Board of Armauer Hansen Research Institute (AHRI)/All Africa Leprosy Education, Research, and Training Center ethics review committee (AF-10-015.1, PO/07/19). We obtained informed written consent from all participants and guardians or parents for minors.

#### Recruitment of participants

Patients with (history within 48 h) fever that presented at the two health facilities and tested positive for malaria by microscopy were recruited as index cases (index) from April to July 2022. We recruited febrile patients who attended the same clinic and tested negative for malaria as controls within 72 h of when the index was identified. The index and controls were followed to their homes, and their household/dormitory members were tested for malaria and their households/dormitories were screened for *Anopheles* mosquitoes (larvae and adult). Household/dormitory members of cases and controls who were willing to participate in the reactive case detection were included irrespective of their symptoms. Households were surveyed for mosquitoes when the head of the household and members of the dormitory gave consent to allow the study team to use mosquito collection methods in their houses/dormitories. Families of cases or controls who were not available within 72 h of recruitment of the cases or controls irrespective of their symptoms were excluded as well as individuals or family members who were unwilling/refused to give informed written consent. It is noticeable that, although the study was unmatched due to the difficulty in recruiting matched controls in geographical proximity of the cases, their general characteristics were very similar. Detailed characteristics of study participants are presented in Table 1.

#### Sample size

We planned an unmatched case:control ratio of approximately 1:2 (ref. ^56^) with prospective case identification until the stopping rule was achieved. The choice of the case:control ratio was based on a logistic regression model aimed to detect an OR of at least 2, assuming an exposure of 20% in controls at household level, where the exposure was defined as presence of *An. stephensi*. The power analysis was conducted in epiR package (R-cran software), and the stopping rule was set to a power of 70% for the study to be sufficiently powered to detect differences between the presence of malaria on *An. stephensi* exposure at household level. The controls were selected from the same population as the cases and post-stratification applied. Data from cases and controls were reviewed regularly, and final sample size was set to 290 with 101 cases and 189 controls. The recruitment of case household and control household members was done to include reactive case detection and improve the power of the study (as well as the OR minimum detection).

#### Data collection

Data on the sociodemographic, epidemiological, intervention and travel history were collected verbally using pre-tested questionnaires which were uploaded to mobile tablets using REDCap tools. The entomological survey data and intervention availability were scored by the study data collectors. Malaria case incidence data (from January 2019 to May 2022) were collected from the records of both private and public health facilities (*n* = 34).

#### Blood samples collection

Finger prick blood samples (~0.5 ml), collected in BD K_2_EDTA Microtainer tubes, were used to diagnose malaria using RDT (Abbott Bioline Malaria Ag Pf/Pv HRP2/LDH) and microscopy, and to prepare dried blood spots (DBS) on 3MM Whatman filter paper (Whatman). The remaining blood was separated into cell pellet and plasma. Slide films were confirmed by expert microscopists. Sociodemographic, epidemiological, intervention utilization, and history of travel and malaria were collected from all study participants.

#### Entomological surveys

We surveyed immature stages of *Anopheles* mosquitoes within a 100-m radius of the index and control houses/dormitories targeting both manmade water storage containers and natural habitats including riverbeds and stream edges. We checked each aquatic habitat for 10 min from 9:00 to 11:00 and 15:00 to 17:00 for the presence of *Anopheles* mosquitoes’ larvae or pupae aiming for ten dips per habitat (using a standard dipper with 350 ml capacity). Characteristics of water holding containers (permanency of habitat, lid status, purpose, volume, presence of shade, type, turbidity, temperature and water source) were recorded for each habitat (Extended Data Table 6). We searched adult mosquitoes using Prokopack aspirators for 10 min between 6:00 and 8:00 indoor, outdoor and in animal shelters located within the compound of the household or inside and outside the dormitories at the university (Extended Data Table 7). Mosquito surveys (immature and adult) were done within 48–72 h of when the index/control was recruited.

Conventional adult mosquito collection methods such as Centers for Disease Control and Prevention light traps and pyrethrum spray sheet have limited sensitivity for this invasive species mainly related with its unique resting behavior^21^. To supplement the evidence generated from the case–control study and examine the resting sites of the adult *Anopheles* mosquitoes in detail in the study area, additional adult mosquito surveys were done targeting potential resting sites including animal shelters and manholes within the study time and area. Informed by these preliminary findings, surveys were systematized in three fortnightly rounds during the study period. In the city, households with (*n* = 15) and without (*n* = 15) animal shelters were included (Extended Data Table 7). At DDU, two dormitory buildings which reported the highest number of malaria cases and their surroundings were selected. Adult mosquitoes were surveyed indoor, outdoor, in animal shelters, in overhead tanks and in manholes using Prokopack aspirators for 10 min between 6:00 and 8:00. Animal shelters were not available at DDU. Adult-caught mosquitoes (sorted on the basis of their abdominal status), and those raised from aquatic stages, were morphologically identified to the species level^22^ (Extended Data Table 8). *Anopheles* mosquitoes were individually preserved in tubes that contained silica gel desiccant in zipped bags and transported to the lab at the AHRI for further analysis. The global positioning system (GPS) coordinates of the households and immature and adult mosquito collection sites were recorded using GARMIN handheld GPS navigator (GARMIN GPSMAP 64S).

### Laboratory procedures

#### Nucleic acid extraction from whole blood and parasite quantification, and genotyping

Blood samples in ethylenediaminetetraacetic acid (EDTA) tubes were used to extract genomic DNA using MagMAX magnetic bead-based technology DNA multi-sample kit on KingFisher Flex robotic extractor machine (Thermo Fisher Scientific). Fifty microliters of whole blood input was eluted in a 150 μl low-salt elution buffer. Multiplex qPCR targeting the 18S rRNA small subunit gene for *P. falciparum* and *P. vivax* was run using primer and probe sequences described by Hermsen^57^ and Wampfler^58^ using TaqMan Fast Advanced Master Mix (Applied Biosystems). *P. falciparum* parasites were quantified using standard curves generated from a serial dilution of NF54 ring stage parasites (10^6^ to 10^3^ parasites ml^−1^). For *P. vivax*, parasite quantification was done using plasmid constructs to infer copy numbers by running serial dilutions (10^7^ to 10^3^ copies µl^−1^) of plasmids having the amplicon. Serial dilutions of the standard curves were generated in duplicate on each plate. Multiplexed amplicon sequencing was performed on qPCR-positive samples with reagents and protocol as in Tessema et al.^59^. DNA was amplified for 15 or 20 cycles in multiplexed PCR, depending on parasitemia and ability to amplify, and for 15 cycles for indexing PCR. The primer pools used in this study comprised high-diversity microhaplotype targets (*n* = 162), polymorphisms associated with drug resistance, and targets in and adjacent to *pfhrp2* and *pfhrp3* to assess for gene deletion (Primer pools 1A and 5 as described in protocols.io repository)^60^. Amplified libraries were sequenced in a NextSeq 2000 or a MiniSeq instrument using 150PE reads with 10% PhiX.

#### Nucleic acid extraction from mosquitoes, assessment of infectivity and bloodmeal source and confirmation of morphological species identification

Infection detection in wild caught mosquitoes is commonly based on an enzyme-linked immunosorbent assay (ELISA)-based protocol that targets circumsporzoite protein (CSP) that is expressed on the surface of *Plasmodium* sporozoites. Low-level expression of CSP at stages of sporogony before the parasites migrate to the salivary gland might interfere with signal detected^61^. Several studies have reported false positive results when targeting CSP especially in zoophilic mosquitoes^62,63^. The false positive results could lead to an overestimation of mosquito infection rates. To achieve a conservative estimate of mosquito infection rates, we implemented stringent steps as indicated below:

1. **Bisected mosquitoes**: We observed previously^61^ that a signal detected from an earlier stage of sporogony might interfere with interpretation of sporozoite detection, probably causing false positive results. We bisected the mosquitoes anterior to the thorax–abdomen junction under a stereo microscope before processing them for infection detection^64^. The head and thoraces were processed and stored separately from the abdomen of the mosquitoes. We only used the head and thorax part for infection detection following homogenization in a robust semi-high-throughput mini-bead beater protocol we developed previously^65^. The heads and thoraces of the mosquitoes were homogenized in 150 µl molecular-grade water that contains 0.2 g zirconium bead (1 mm diameter) using a Mini-Bead Beater 96. Part of the homogenate (50 µl) was used for nucleic acid extraction using cetyl trimethyl ammonium bromide^62^; 100 µl grinding buffer (0.5% w/v case in, 0.1 N NaOH in 10 mM PBS, pH 7.4, and 0.5% IGPAL CA-630) was added to the remaining that was used to screen samples for circumsporozoite in bead-based assay.
2. **Circumsporozoite bead-based assay**: We adopted the most advanced (highly sensitive) bead-based assay for infection detection in mosquitoes^66^ by targeting CSP. Antibody-coupled magnetic beads and biotinylated secondary antibodies were obtained from the Centers for Disease Control and Prevention, Division of Parasitic Diseases and Malaria, Entomology Branch, Atlanta, GA, USA, and implemented as described before^7^ and were run using MagPix immunoanalyzer (Luminex Corp, CN-0269-01).
3. **Quality control to reduce cross-reactivity**: The bead-based assay we adopted may eliminate false negatives due to lower limit of detection than previous ELISA-based assays^66^ but also brings a challenge of enhanced detection of cross-reacting proteins. To reduce this chance, mosquito homogenate was boiled at 100 °C before processing to eliminate false positives that may be caused by heat-unstable cross-reactive proteins to strengthen the validity of the results. To ascertain this specificity issue, we have included colony-maintained *An. arabiensis* and *An. stephensi* mosquitoes fed on sugar solution and patients’ blood in direct membrane feeding assays (had infection status determined morphologically in the same mosquito batches) that were used as negative and positive controls, respectively. *Plasmodium*-infected mosquitoes were used as positive controls along with sugar-fed mosquitoes as negative controls in every extraction round (Supplementary Fig. 3 and Supplementary Tables 1 and 2).
4. **Retesting and confirmatory 18S-based species-specific PCR**: Samples with higher mean fluorescence intensity signal than the negative controls plus three standard deviations and a representative set of mosquitoes that gave low signal were rerun to confirm the observations. Genomic DNA extracted from the head and thoraces of all mosquitoes was tested on a PCR that targeted 18S small ribosomal subunit gene as a confirmatory test. Only mosquito samples positive by the CSP-based assays and 18S-based PCR were considered infected.

Nucleic acid was extracted from the abdomen of fully engorged mosquitoes for bloodmeal source identification following the same procedure used for the head and thoraces using a cetyltrimethylammonium bromide (CTAB)-based method as described before^67^. A multiplex PCR assay that amplifies the cytochrome b gene based on Kent and Norris^68^ was used for bloodmeal source analysis. We have introduced slight modifications to improve product size separation on gel electrophoresis. The multiplex of cow and dog was separately done from the multiplex of goat and human. The optimized PCR thermal cycler conditions were: 5 min at 95 °C as an initial denaturation followed by 40 cycles of denaturation at 95 °C for 60 s, annealing at 56 °C for 60 s for cow and dog multiplex, and 62 °C for goat and human multiplex, followed by an extension at 72 °C for 60 s, and 1 cycle of the final extension at 72 °C for 7 min.

Confirmation of the *Anopheles* morphological identification was done following a recently published protocol that targets the *ITS2* gene^69^. *An. stephensi* diagnostic amplicon of 438 bp size was expected while a universal amplicon of varying sizes (>600 bp), depending on the length of *ITS2* in a particular species, was expected in this multiplex protocol (Supplementary Fig. 4). The universal amplicon was used to serve as an internal control to rule out PCR failure.

### Data management and analysis

#### Data management

Study data collection tools (mobile application version 5.20.11) were prepared and managed using REDCap electronic data capture tools hosted at AHRI. CSV files exported from REDCap were analyzed using STATA 17 (StataCorp), RStudio v.2022.12.0.353 (Posit, 2023), QGIS v.3.22.16 (QGIS Development Team, 2023, QGIS Geographic Information System, Open Source Geospatial Foundation Project), GraphPad Prism 5.03 (GraphPad Software) and RStudio using packages lme4 (generalized linear mixed models) and dcifer^70^ (pairwise relatedness analysis on *P. falciparum* genotypes in diverse loci).

#### Description of study variables

We collected the following variables in this study:

- **Sociodemographic:** sex, age, educational level and occupation
- **Household characteristics:** main materials used for building the household, fuel source, water source and presence of water bodies near the household/dormitory, and presence of livestock
- **Intervention:** presence, number, and condition of bed nets, use of bed nets, use of smoke repellents or aerosol mosquito spray, and history of insecticide residual spray
- **Diagnosis and treatment:** malaria test result by RDTs and microscopy, temperature, presence of symptoms and treatment history, and pregnancy status
- **Human behavior:** travel history, health seeking behavior, sleeping and waking time, and sleeping place
- **Entomological survey:** mosquito collection method and time of collection, mosquito species detected and density, *Anopheles* species detected and density, abdominal status of mosquitoes detected, type of aquatic habitat near the household/dormitory, and type and characteristics of water sources detected within 100-m radius around the household/dormitory

#### Bioinformatic analysis

FASTQ files from multiplexed amplicon sequencing of *P. falciparum* were subjected to filtering, demultiplexing and allele inference using a Nextflow-based pipeline^71^. We used cut adapt to demultiplex reads for each locus based on the locus primer sequences (no mismatches or indels allowed), filter reads by length (100 base pairs) and quality (default NextSeq quality trimming). We used dada2 to infer variants and remove chimeras. Reads with a Phred quality score of less than 5 were truncated. The leftmost base was trimmed and trimmed reads of less than 75 base pairs were filtered out. Default values were used for all other parameters. We then aligned alleles to their reference sequence and filtered out reads with low alignment. We masked homopolymers and tandem repeats to avoid false positives.

#### Genetic analysis

Pairwise relatedness analysis was performed on *P. falciparum* genotypes in diverse loci using Dcifer with default settings^70^. Pairwise relatedness was only considered between samples where the lower 95% CI of estimated relatedness was greater than 0.1. Point estimates of pairwise relatedness that satisfied this threshold were then binned into low, medium and high relatedness at greater than 0.2, 0.5 and 0.9 respectively. Samples were then clustered based on pairwise relatedness. Drug resistance marker genotypes were extracted from loci of interest. Evidence of *pfhrp2* and *pfhrp3* deletions were identified from a drop in normalized coverage in amplicons within and surrounding *pfhrp2* and *pfhrp3*. Complexity of infection was estimated by taking the 0.97 quantile (fifth highest number) of observed alleles across loci to minimize the impact of false positives on estimates.

#### Epidemiological analysis

We used standard case–control analyses to examine the association between risk factors and malaria infection. It calculates point estimates and CIs for the OR along with the significance level based on the chi-squared test. Continuous variables were presented as median and IQR. Tests of association between two categorical variables were performed using chi-squared test on contingency tables. Mann–Kendall statistical test was used to test for monotonic (increasing or decreasing) trends of malaria cases using the secondary data obtained from the private and public health facilities at the city and DDU.

#### Spatial data analysis

As the dormitories within the university study site were located within a small area (20 buildings in 45,450 m^2^ area), clustering of prevalence data was assessed in the city only. The prevalence of malaria by RDT and/or microscopy was calculated for each household. Global and local Moran’s *I* calculations were used to estimate the level of spatial autocorrelation within household prevalence data. The statistical strength of association for global Moran’s *I* was calculated using Monte-Carlo methods based on 9,999 times permutations of the prevalence data. The Euclidean distance from the river to every site where adult or larval *An. stephensi* were located were calculated in meters.

#### Statistical analysis

To identify the association of *An. stephensi* and other risk factors for malaria positivity and quantify the variation in a parasite positive outcome in Dire Dawa, we employed a multilevel logistic regression model with nested random effects (heterogeneous household and case–control group variances) to account for intra-class correlation^72^. The covariates included for the multi-level logistic regression analysis with random effect are listed in detail in Supplementary Table 3. Having more than 30 potential covariates associated to malaria, more than one billion models for exhaustive best model searching (excluding interactions between covariates), we reduced the number of covariates to a manageable size by considering univariate generalized mixed models (with case index as random effect instead of setting which were not contributing to the differences in malaria positivity for cases and controls) and considering only the covariates with *P* value lower than 0.3 within these models (Supplementary Table 3). The decision to use case/control as random effect instead of fixed effect came from preliminary analysis that considered the best candidate(s) for random effects. Variable selection was performed by testing 2,000+ binomial logistic mixed models (number of tested models depending on initial screening). During the initial screening, a candidate variable was selected if its *P* value, obtained from a Wald test applied to the variable’s estimated coefficient in logistic regression, was lower than 0.3. The models were ranked on the basis of their Akaike Information Criteria (AIC) and the Bayes information criteria (BIC) values, with the top model being the one with the lowest AIC value^73^. Variable selection was repeated for three different response variables: model 1 with response RDT/microscopy, model 2 with response RDT/microscopy/qPCR, and, finally, model 3 with response qPCR. As a result, only five of the 12 factors assessed for individual and household characteristics (sex, age, *An. stephensi* larvae and/or adult presence, natural waterbody existence, and use of aerosol insecticide spray) were included for the final model (Supplementary Table 4). We also explored interactions between gender, age and site.

After model selection with several model outcomes and distribution (Supplementary Table 4), the binomial model with outcome represented by malaria positivity (positive/negative) using RDT and/or microscopy best represented the relationship between malaria and risk factors (Supplementary Table 4)^74^. In this model, the employment of geographic unit’s effects such as household and area setting (city versus university) enabled us to control for unknown variations by including them as random effects in the model. In fact, individuals living in the same household may share exposures that can determine similarities in malaria transmission as well as in the larger setting (city versus university).

Let *y*_*ij*_ denote the malaria outcome of the *i*th individual in the *j*th household or cluster, identified by the RDT and/or microscopy with probability *π*_*ij*_ where *y*_*ij*_ = 1 denotes the individual tested positive, while *y*_*ij*_ = 0 denotes the individual tested negative for malaria. A multilevel logistic regression model with random effects for the outcome *y*_*ij*_ is given by

$$\log it({\pi }_{ij})={\beta }_{0j}+\beta {X}_{ij}+{u}_{j}$$

where *X*_*ij*_ = (1, *x*_1*ij*_,…, *x*_*pij*_) is vector of *p* explanatory variables or covariates measured on the *i* individual and on the *j* household (cluster), *β* is vector of fixed regression coefficients or parameters and *u*_*j*_ is a random effect varying over household and case control.

### Reporting summary

Further information on research design is available in the Nature Portfolio Reporting Summary linked to this article.

## Online content

Any methods, additional references, Nature Portfolio reporting summaries, source data, extended data, supplementary information, acknowledgements, peer review information; details of author contributions and competing interests; and statements of data and code availability are available at 10.1038/s41591-023-02641-9.

### Supplementary information

Supplementary Information Supplementary information.

Reporting Summary

Source Data Tables 1–3 This file contains the source dataset for the three tables that are part of the main text of the paper. It contains three tabs that are labeled: Source_Data_Table_1, Source_Data_Table_2 and Source_Data_Table_3.

### Source data

Source Data Fig. 1 Global *An. stephensi* distribution (native and invasive range) and detection of *Plasmodium* infection for Fig. 1a and geographic location of the study area (households/dormitories, aquatic habitats and health facilities) for Fig. 1b source data.

Source Data Fig. 2 Source data obtained from the national DHIS2 repository for Dire Dawa between January 2013 and May 2022 and data collected during the study period from the study participants infection status (as measured by RDTs, microscopy and quantitative species-specific 18S-based PCR) in separate Excel sheet for Fig. 2a–e.

Source Data Fig. 3 Source data on the geographic location of infections detected in the study households (Fig. 3a), *Anopheles* mosquito presence (Fig. 3b) and overlap of *Plasmodium* infection detected in study participants and *An. stephensi* in the respective households (Fig. 3c) and pairwise genetic relatedness data (Fig. 3d) and their clustering (Fig. 3e).

Source Data Extended Data Table 1 Source data collected from 34 private and public health facilities located in Dire Dawa for the period between January 2019 and May 2022.

Source Data Extended Data Table 2 Source data on symptom status (axillary temperature ≥37.5 °C) by study site and participant category.

Source Data Extended Data Table 3 Sociodemographic, intervention utilization and malaria predisposing factors related to malaria infection source data.

Source Data Extended Data Table 4 Infection status (as measured by RDTs, microscopy and qPCR) source data.

Source Data Extended Data Table 5 Source data on infection status (as measured by RDTs, microscopy and qPCR as well as parasite density determined by the latter).

Source Data Extended Data Table 6 Source data related to adult and larvae mosquito surveys. Individual adult mosquito data including date, method and place of collection, and abdominal status included.

Source Data Extended Data Table 7 Source data related to adult and larvae mosquito surveys. Individual adult mosquito data including date, method and place of collection, and abdominal status included.

Source Data Extended Data Table 8 Source data related to adult and larvae mosquito surveys. Individual adult mosquito data including date, method and place of collection, and abdominal status included.

Source Data Extended Data Table 9 Source data collected during the study period from the study participants on infection status (as measured by RDTs and microscopy), sociodemographic status, intervention utilization and malaria predisposing factors related to parasite lineages.

## Figures and Tables

**Extended Data Table 1:**
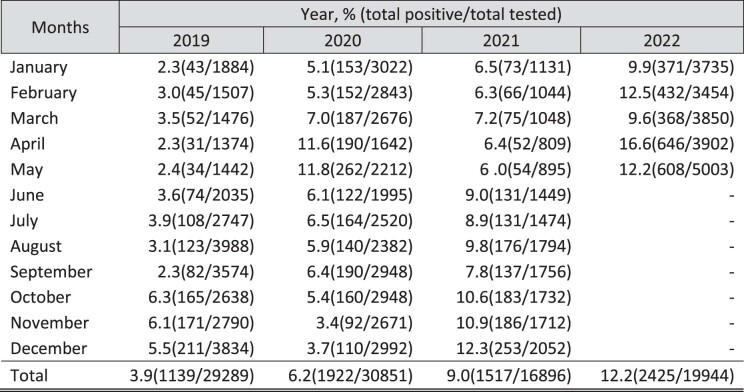
Monthly total cases tested for malaria and positivity rate (determined by microscopy) in Dire Dawa between January 2019 and May 2022, obtained from 34 public and private health facilities

Cases over total tested are shown within brackets.

Source data

**Extended Data Table 2:**
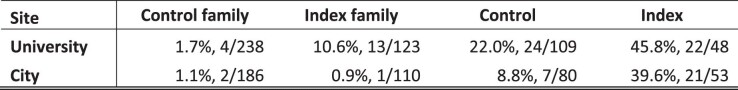
Symptom status (axillary temperature ≥37.5 °C) by study site and participant category

Source data

**Extended Data Table 3:**
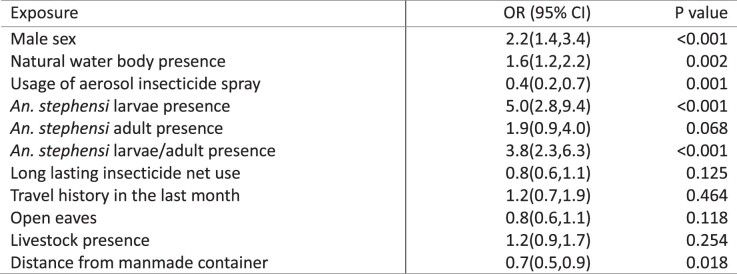
Case control analysis, risk factors associated with the index cases and their family members

Odds ratios were determined from case-control odds ratio analysis. *p* values were estimated from a chi-squared test. OR, odds ratio; 95% CI, 95% confidence interval

Source data

**Extended Data Table 4:**
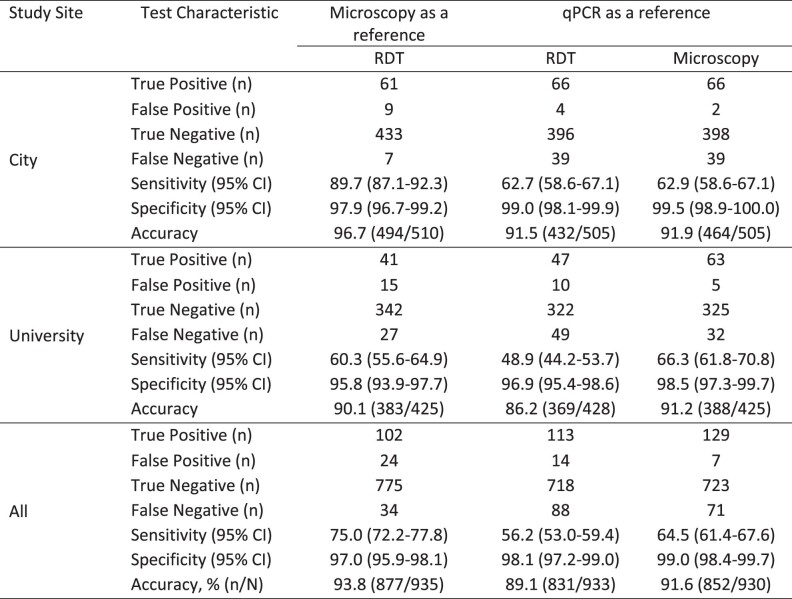
Overall performance of RDT/Microscopy vs qPCR for the diagnosis of malaria detection by study site

RDT, rapid diagnostic test; qPCR, real time PCR targeting 18S gene; 95% CI, 95% confidence interval.

Source data

**Extended Data Table 5:**
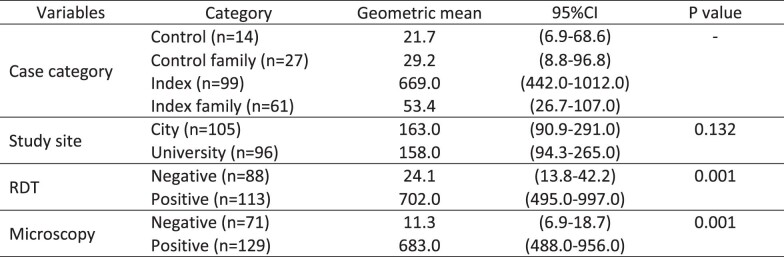
Comparison of mean parasite density between groups

Unpaired Student’s t-test, two-tailed, was used to compare groups. RDT, rapid diagnostic test; 95% CI, 95% confidence interval.

Source data

**Extended Data Table 6:**
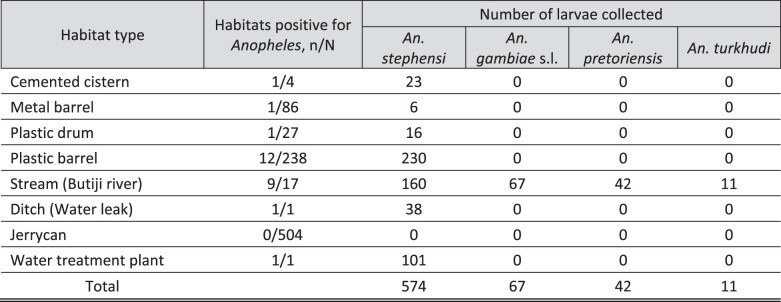
Immature *Anopheles* mosquitoes collected during the case control study within 100-meter radius around the case and control households

Source data

**Extended Data Table 7:**
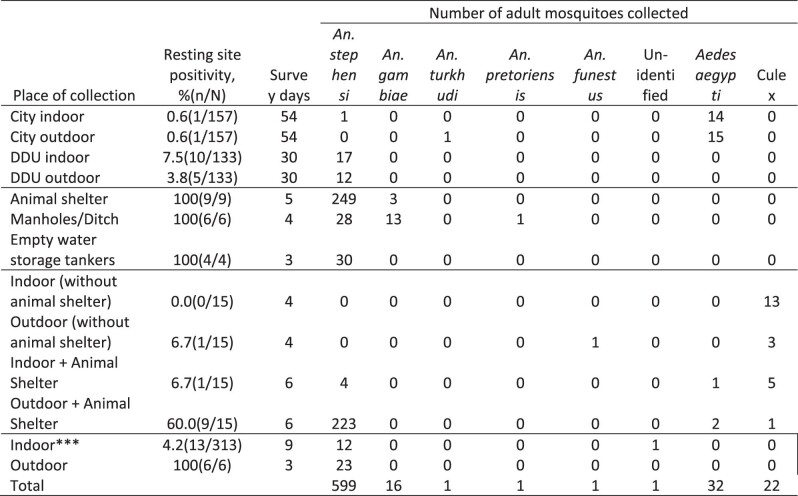
Adult mosquitoes collected in the case control and expanded surveys in Dire Dawa

*Adult mosquitos collected from outside of houses involved in the case control study in Goro and Dire Dawa University (DDU);

**The expanded survey in the city was done in three rounds for each collection place;

***The expanded survey at DDU included an adult mosquito survey in four buildings that were fortnightly sampled.

Source data

**Extended Data Table 8:**
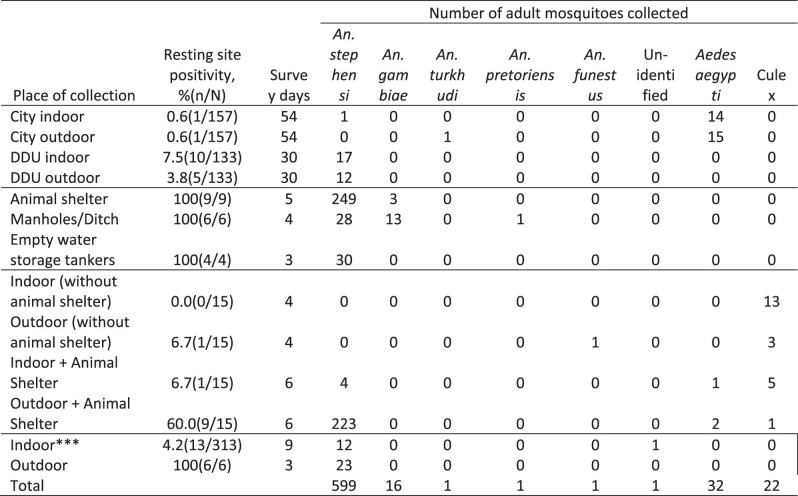
Summary of abdominal status for adult mosquitoes collected during the study period

Counts over total morphologically diagnosed is provided in each cell.

Source data

**Extended Data Table 9:**
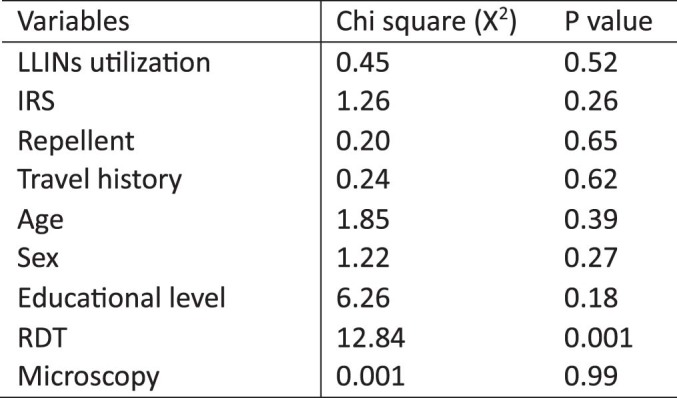
Association of lineages with intervention utilization, travel history and diagnostic outcomes

Source data

## Data Availability

All the data used in the paper are available on dryad (linked with the ORCID: https://orcid.org/0000-0003-1931-1442). Sequence data are deposited on NCBI with the BioProject accession number PRJNA962166. Raw data of the study will be available in the future upon request following signing of data sharing agreement, abiding to institutional and international data sharing guidelines. Source data are provided with this paper.

## Code availability

The R codes used to run the analyses reported in this study can be found at https://github.com/legessealamerie/DD-Stephensi and https://github.com/EPPIcenter/mad4hatter.
