# Supplementary material for: Evidence for a role of Anopheles stephensi in the spread of drug- and diagnosis-resistant malaria in Africa
Source: Nat Med. 2023 Oct 26;29(12):3203–11. doi: 10.1038/s41591-023-02641-9 (PMC10719088; doi:10.1038/s41591-023-02641-9)
Supplement: Supplementary file 1 — Supplementary information. [file 41591_2023_2641_MOESM1_ESM.pdf]

nature medicine

Article

https://doi.org/10.1038/s41591-023-02641-9

Evidence for a role of *Anopheles stephensi* in the spread of drug- and diagnosis-resistant malaria in Africa

In the format provided by the authors and unedited

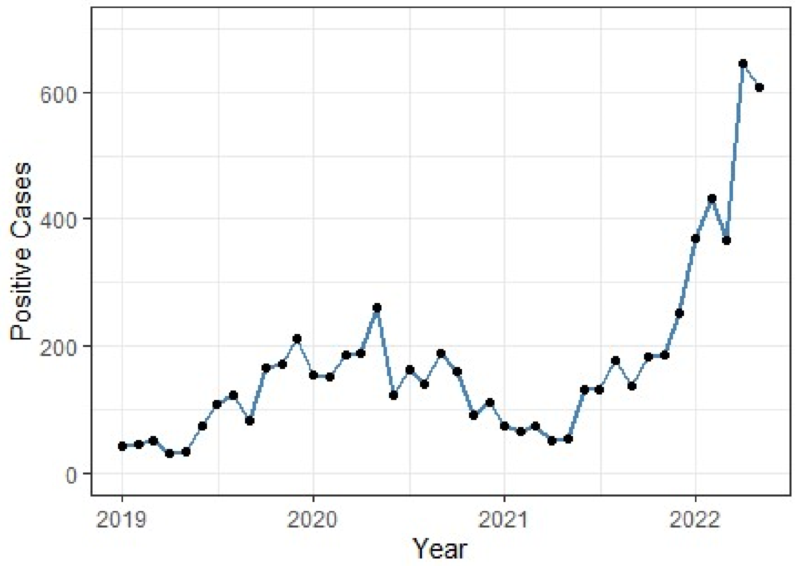

Figure S1: Number of monthly malaria positive cases in Dire Dawa city from January 2019 to May 2022. Data obtained from DHIS2 and collected from the 34 health facilities is only up to May 2022.

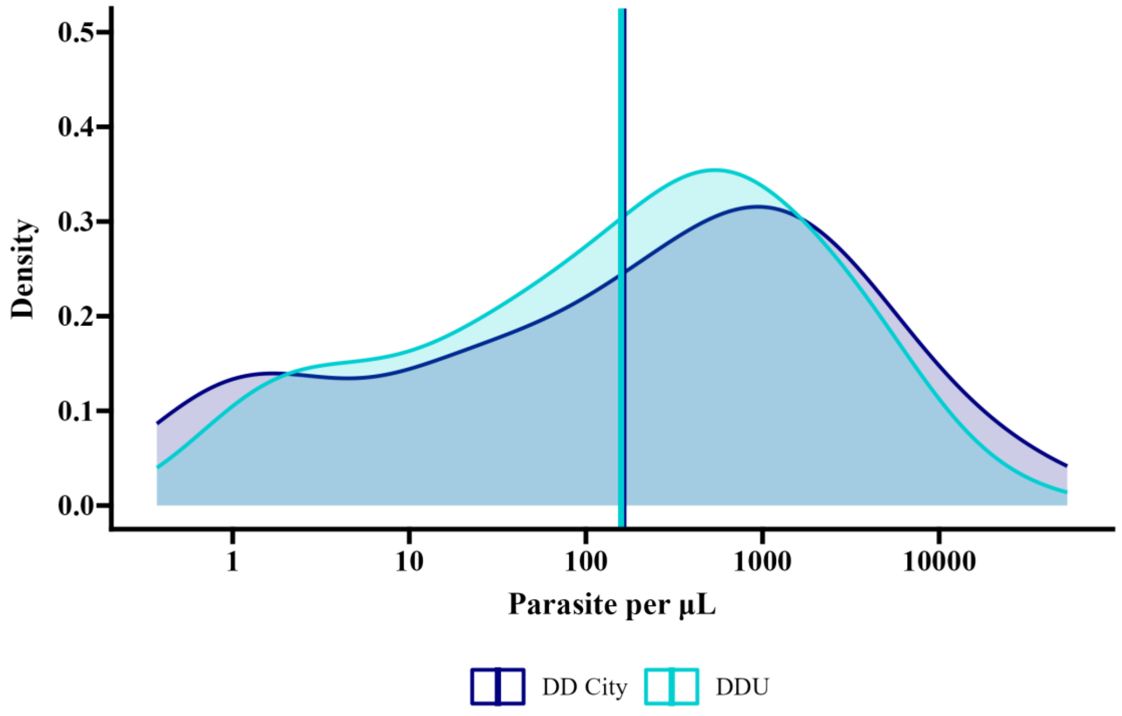

Figure S2: Parasite density distribution between university students and city residents with relative averages.

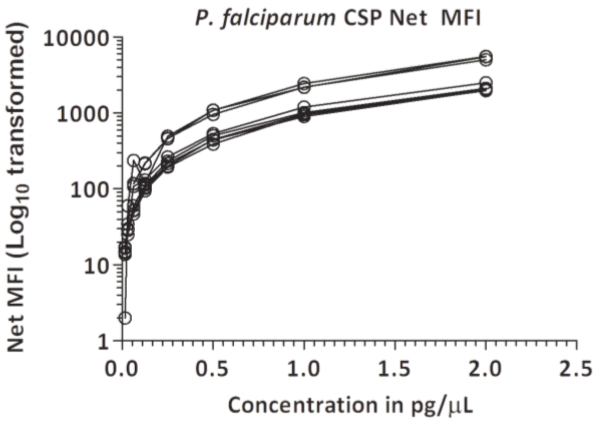

Figure S3: Summary of the standard curves generated using recombinant proteins specific for *P. falciparum*.

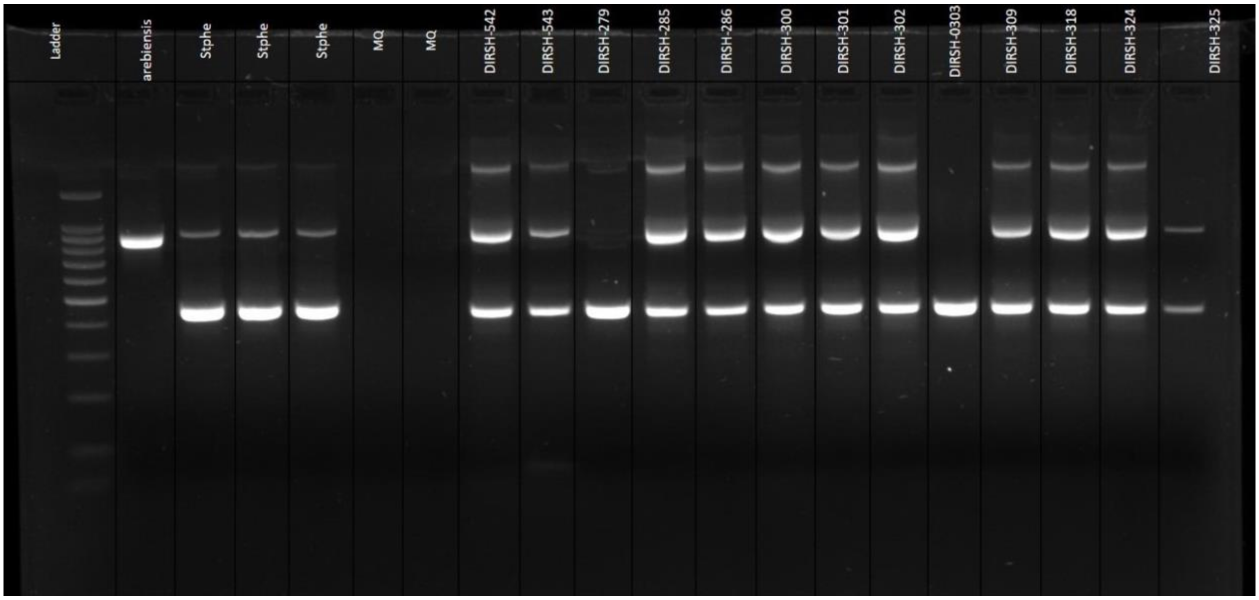

Figure S4: Example gel picture of ITS2 based PCR for confirmation of *An. stephensi*. In these assays, colony *An. arabiensis* (Lane 1) and *An. stephensi* (Lane 2-4) mosquitoes were included as positive extraction and PCR controls and MQ (Lane 5-6) as a negative PCR control together with wild caught morphologically *An. stephensi* identified mosquitoes (Lanes 7-19). The first lane to the left is a 100bp molecular weight marker (labelled Ladder). The experiment outcomes were consistent with morphological outcomes. We did not repeat the PCR experiments for species identification.

Table S1: Net Mean Fluorescence Intensity (MFI) values of recombinant proteins and positive control mosquitoes

**Recombinant protein**	**Concentration in pg/µL**	**Net MFI of First Batch Experiments**				
		Exp't 1	Exp't 2	Exp't 3	Exp't 4	Exp't 5
P. falciparum CSP recombinant protein 2-fold serial dilution used for the validation of CSP bead-based assay	2	1,975.8	2,498.5	2,110.5	2,073.0	1,982.5
	1	948.8	1,202.0	1,008.5	970.5	904.0
	0.5	390.8	533.0	503.0	447.5	441.5
	0.25	193.8	265.0	232.5	200.0	216.0
	0.125	94.3	131.0	112.5	102.0	105.5
	0.0625	46.8	108.0	60.0	52.0	52.5
	0.03125	29.8	34.5	29.5	25.0	28.5
	0.015625	13.8	17.0	16.5	15.0	14.5
Positive colony mosquitoes used as extraction and CSP quality control		281.0	306.0	24,376.5	24,560.0	24,043.5
		19,314.0	19,005.0	21,203.5	24,234.0	26,086.5

MFI, mean fluorescence intensity; Exp't #, experiment number; N/A, not available; CSP, circumsporozoite protein

Table S2: Net Mean Fluorescence Intensity (MFI) values of negative adult *An. stephensi* mosquitoes reared from larvae used as a negative control

Exp't #	**ID**	**Pf**
Exp’t #01	NC-01	0.8
Exp’t #01	NC-02	0.8
Exp’t #01	NC-03	0.8
Exp’t #01	NC-04	0.8
Exp’t #02	NC-01	1.0
Exp’t #02	NC-02	1.0
Exp’t #02	NC-03	0.0
Exp’t #02	NC-04	1.0
Exp’t #03	NC-05	4.5
Exp’t #03	NC-05	3.5
Exp’t #03	NC-05	4.0
Exp’t #04	NC-05	9.0
Exp’t #04	NC-04	6.0
Exp’t #04	NC-04	3.0
Exp’t #04	NC-04	2.0
Exp’t #05	NC	2.5
Exp’t #05	NC	2.5
Exp’t #05	NC	1.5
Exp’t #05	NC	2.5
Exp’t #05	NC	2.5
Exp’t #05	NC	2.5
Exp’t #05	NC	4.5
Exp’t #05	NC	5.5
Exp’t #05	NC	2.0
Exp’t #05	NC	1.5
Exp’t #05	NC	1.5
Exp’t #05	NC	4.5
Exp’t #06	NC (DDL-232)	2
Exp’t #06	NC (DDL-499	14
Exp’t #06	NC (DDL-232)	11
Exp’t #06	NC (DDL-233)	2
Exp’t #06	NC (DDUIC-025 #6-2)	1
	average	3
	SD	3
	average + 3*SD	**12.5**

NC, negative control; Exp’t #, experiment number; SD, standard deviation; Pf, *P. falciparum*

Table S3: Variable selection results.

	Full data	City	University
**Screened variables (*p* value <0.3).**	Site, larvaepos, larvaespp, larvaesteph, adultsppindoor, adultsppoutdoor, dist_river, SEX, REP, IRS, LLIUSE, stephpos	Stephpos, latitude, adultsppindoor, adultsppoutdoor, dist_river, SEX, AGE, REP, LLINLN, WT12, EDU	Stephpos, longitude, larvaepos, larvaespp, adultsppindoor, adultsppoutdoor, travelprop, AGE, EAVE, LLIH, WT12
**Number of models tested**	4096	2048	2048
**Min AIC**	431	213	198
**Max AIC**	598	260	210

**Variables in the top 10% of models (Contributing to at least 20% of the top 10%)**			
	Distance river (83%)	Distance river (98%)	Longitude (97%)
	Site (45%)	Stephensi positive (49%)	Larvae positive (64%)
	Sex (27%)	Sex (48%)	Stephensi positive (44%)
	Larvaepos (25%)	Education (20%)	Larvae Stephensi (25%)
	Adult spp outdoor (22%)	Adult spp outdoor (20%)	
	Larvae Stephensi (20%)		

Table S4: Results from a multi-level logistic regression model

**Factors**	**Category**	**Fitted models**					
		**Model 1**		**Model 2**		**Model 3**	
		**Estimate (SE)**	**P value**	**Estimate (SE)**	**P value**	**Estimate (SE)**	**P value**
Sex	Female (Ref.)						
	Male	1.1 (0.3)	<0.001	0.6 (0.3)	0.044	0.6 (0.3)	0.034
Age in years	< 5 years (Ref.)						
	5 -15 Years	1.3 (0.7)	0.071	0.4 (0.6)	0.509	0.6 (0.6)	0.358
	Above 15 Years	1.5 (0.7)	0.029	0.8 (0.5)	0.126	1.0 (0.6)	0.063
An. stephensi larvae and/or adult presence	Absent (Ref.)						
	Present	1.2 (0.4)	<0.001	1.6 (0.4)	<0.001	1.5 (0.4)	<0.001
Natural waterbody presence	Absent (Ref.)						
	Present	0.6 (0.3)	0.089	0.4 (0.4)	0.241	0.5 (0.4)	0.149
Usage of aerosol insecticide spray	Not Using (Ref.)						
	Using	-1.3 (0.5)	0.016	-1.6 (0.6)	0.005	-1.4 (0.5)	0.007
**Fit Statistics**							
-2log(likelihood)		755.2		859.8		833.8	
AIC (smaller is better)		775.4		879.9		853.9	
BIC (smaller is better)		823.3		927.7		901.7	

Estimated regression coefficients (SE), *p*-value from Wald test and fit statistics for the multi-level logistic regression model.
