# Supplementary material for: Evidence for a role of Anopheles stephensi in the spread of drug- and diagnosis-resistant malaria in Africa
Source: Nat Med. 2023 Oct 26;29(12):3203–11. doi: 10.1038/s41591-023-02641-9 (PMC10719088; doi:10.1038/s41591-023-02641-9)
Supplement: Supplementary file 2 — Reporting Summary [file 41591_2023_2641_MOESM2_ESM.pdf]

nature portfolio

Corresponding author(s): Fitsum G Tadesse

Last updated by author(s): 2023/10/04

Reporting Summary

Nature Portfolio wishes to improve the reproducibility of the work that we publish. This form provides structure for consistency and transparency in reporting. For further information on Nature Portfolio policies, see our Editorial Policies and the Editorial Policy Checklist.

Statistics

For all statistical analyses, confirm that the following items are present in the figure legend, table legend, main text, or Methods section.

n/a	Confirmed	
☐	☒	The exact sample size (*n*) for each experimental group/condition, given as a discrete number and unit of measurement
☐	☒	A statement on whether measurements were taken from distinct samples or whether the same sample was measured repeatedly
☐	☒	The statistical test(s) used AND whether they are one- or two-sided<br>*Only common tests should be described solely by name; describe more complex techniques in the Methods section.*
☐	☒	A description of all covariates tested
☐	☒	A description of any assumptions or corrections, such as tests of normality and adjustment for multiple comparisons
☐	☒	A full description of the statistical parameters including central tendency (e.g. means) or other basic estimates (e.g. regression coefficient) AND variation (e.g. standard deviation) or associated estimates of uncertainty (e.g. confidence intervals)
☐	☒	For null hypothesis testing, the test statistic (e.g. *F*, *t*, *r*) with confidence intervals, effect sizes, degrees of freedom and *P* value noted<br>*Give P values as exact values whenever suitable.*
☒	☐	For Bayesian analysis, information on the choice of priors and Markov chain Monte Carlo settings
☐	☒	For hierarchical and complex designs, identification of the appropriate level for tests and full reporting of outcomes
☒	☐	Estimates of effect sizes (e.g. Cohen's *d*, Pearson's *r*), indicating how they were calculated

Our web collection on statistics for biologists contains articles on many of the points above.

Software and code

Policy information about availability of computer code

Data collection	Study data collection tools (mobile application version 5.20.11) were prepared and managed using REDCap electronic data capture tools
Data analysis	STATA 17 (StataCorp.,TX,USA), Rstudio v.2022.12.0.353(Posit,2023), QGIS v 3.22.16 (QGIS Development Team,2023. QGIS Geographic information system. Open source Geo spatial Foundation Project), and Graph pad prism 5.03 (Graph Pad Software inc., CA, USA). Rstudio v.2022.12.0.353(Posit,2023) was used , with packages lme4 (generalized linear mixed models) and dcifer (pairwise relatedness analysis on P. falciparum genotypes in diverse loci). The R codes can be found at https://github.com/legessealamerie stephensi_outbreak_DireDawa_ETH_For_Publication.

For manuscripts utilizing custom algorithms or software that are central to the research but not yet described in published literature, software must be made available to editors and reviewers. We strongly encourage code deposition in a community repository (e.g. GitHub). See the Nature Portfolio guidelines for submitting code & software for further information.

Data

Policy information about availability of data

All manuscripts must include a data availability statement. This statement should provide the following information, where applicable:

- Accession codes, unique identifiers, or web links for publicly available datasets
- A description of any restrictions on data availability
- For clinical datasets or third party data, please ensure that the statement adheres to our policy

All the data used in the manuscript are available on dryad (linked with the ORCID: https://orcid/0000-0003-1931-1442). Sequence data are deposited on NCBI with the BioProject accession number PRJNA962166. Raw data of the study will be available in the future upon reasonable request. The R codes used to run the analyses reported in this study can be found at https://github.com/legessealamerie/stephensi_outbreak_DireDawa_ETH_For_Publication and https://github.com/EPPIcenter/mad4hatter.

Research involving human participants, their data, or biological material

Policy information about studies with human participants or human data. See also policy information about sex, gender (identity/presentation), and sexual orientation and race, ethnicity and racism.

Reporting on sex and gender	We followed the guidance on sex and gender reporting of the journal. Sex was self-reported in this study. We presented data disaggregated by sex when analyzed malaria risk in population. Individual level data included in the link provided contains sex as a variable at an individual level. Sex or gender was not considered in the study design. The research findings in this study apply in box sexes. Since the outbreak in the university campus happened at a fine spatial scale the dormitories affected by malaria were occupied by male students only. In the city, we had a predictable mix of sex. In the city 240 male and 282 females participated in the study whilst all participants in the university (n=249) were male students. Sex based malaria risk was analyzed in this study for the population in the city. Consent was obtained to share data not linked with personal identifiers.
Reporting on race, ethnicity, or other socially relevant groupings	N/A
Population characteristics	The median age in the city was 23 years (interquartile range 12-35) and in the university it was 22 years(20-22).
Recruitment	Patients with (history in 48 hours) fever that presented at the two health facilities and tested positive for malaria by microscopy were recruited as index cases (index) from April to July 2022. Febrile patients who attended the same clinic and tested negative for malaria were recruited as controls within 72 hours of when the index was identified. The index and controls were followed to their homes and their households/dormitory members were tested for malaria and their households or dormitories were screened for anopheles mosquitoes (larvae and adult). Patients were requested to join the study as far as the capacity of the field team allowed on a first come first served basis.
Ethics oversight	Study protocol was approved by the institutional ethical review board of AHRI/ALERT ethics review committee (AF-10-015.1,PO/07/19). We provided a statement of informed consent in the manuscript.

Note that full information on the approval of the study protocol must also be provided in the manuscript.

Field-specific reporting

Please select the one below that is the best fit for your research. If you are not sure, read the appropriate sections before making your selection.

☒ Life sciences ☐ Behavioural & social sciences ☐ Ecological, evolutionary & environmental sciences

For a reference copy of the document with all sections, see nature.com/documents/nr-reporting-summary-flat.pdf

Life sciences study design

All studies must disclose on these points even when the disclosure is negative.

Sample size	We planned an unmatched case control ratio 1:2 with prospective case identification until stopping rule was achieved. The choice of the ratio was based on a logistic regression model aimed to detect an odds ratio (OR) of at least 2, assuming an exposure of 20 % in controls at household level, where the exposure was defined as the presence of An. stephensi. The power analysis was conducted in epiR package (R-cran software) and stopping rule was set to a power of 70% for the study to be sufficiently powered to detect differences between the presence of malaria on an. An. stephensi exposure at household level. The controls were selected from the same population as the cases post-stratification applied. Data from cases and controls were reviewed regularly, and final sample size was set to 290 with 101 cases and 189 controls. The recruitment of case-household and control-household members was done to include reactive case detection and improve the power of the study (as well as the OR minimum detection).
Data exclusions	No data was excluded from the final analysis

Replication	Infection detection in wild caught mosquitoes that relies on an ELISA based protocol that targets circum sporozoite protein (CSP)is one of the highly informative tools to estimate malaria transmission. However several studies reported false positive results when targeting CSP specially in zoophilic mosquitoes. The false positive results could lead to an overestimation of the entomological inoculation rate. To circumvent this, we implemented stringent steps to determine infection status of mosquitoes. One of the approaches we followed is to rerun the bead based assays and test mosquitoes on a confirmatory PCR. Of the mosquitoes that were scored positive (n=4) and tested again all were confirmed to be positive in the rerun. when tested with 18s based PCR, only 3 of these mosquitoes were confirmed to be positive. We thus implemented a stringent criteria for determining infection status of a mosquito was considered infected only when it was found both CSP and 18S PCR positive which ended up to be only 3 infected An. stephensi mosquitoes.
Randomization	N/A: We recruited consecutive patients with (history within 48 hours) fever that presented at the two health facilities and tested positive for malaria by microscopy were recruited as index cases (index) in a 1:2 ratio (one case: two controls) unmatched study design. So randomization is not relevant for our study since we employed a case control study design.
Blinding	Grouping was determined by the infection status as determined by microscopy slide film investigation for the cases and controls. Once the case or control were identified, the investigators test all their members of the family/dormitory irrespective of their symptoms. Due to this blinding of investigators was not relevant to our study.

Reporting for specific materials, systems and methods

We require information from authors about some types of materials, experimental systems and methods used in many studies. Here, indicate whether each material, system or method listed is relevant to your study. If you are not sure if a list item applies to your research, read the appropriate section before selecting a response.

Materials & experimental systems

n/a	Involved in the study
☒	☐ Antibodies
☒	☐ Eukaryotic cell lines
☒	☐ Palaeontology and archaeology
☒	☐ Animals and other organisms
☒	☐ Clinical data
☒	☐ Dual use research of concern
☒	☐ Plants

Methods

n/a	Involved in the study
☒	☐ ChIP-seq
☒	☐ Flow cytometry
☒	☐ MRI-based neuroimaging
